# Molar Incisor Hypomineralization: Awareness among Postdoctoral Dental Residents: A Cross-Sectional Study

**DOI:** 10.3390/dj10040064

**Published:** 2022-04-06

**Authors:** Jana Negrescu, Laurenc Kodra, Hassan Ziada, Tanya Al-Talib, Neamat Hassan Abubakr

**Affiliations:** 1 DMD Students School of Dental Medicine, University of Nevada, Las Vegas, NV 89557, USA; negrej1@unlv.nevada.edu (J.N.); kodra@unlv.nevada.edu (L.K.); 2 Department of Clinical Sciences, School of Dental Medicine, University of Nevada, Las Vegas, NV 89557, USA; hassan.ziada@unlv.edu (H.Z.); tanya.al-talib@unlv.edu (T.A.-T.); 3 Department of Biomedical Sciences, School of Dental Medicine, University of Nevada, Las Vegas, NV 89557, USA

**Keywords:** molar incisor hypomineralization, dental education, dental post-doctorate residence

## Abstract

Background: Molar incisor hypomineralization (MIH) is the presentation of an enamel defect, where incisors and one (or more) molars are affected. Identifying MIH is significant in restoring its visual defect and avoiding pain or other consequences of this condition. The present cross-sectional study aimed to evaluate the awareness, ability, and confidence in identifying MIH among postgraduate residents in the state of Nevada. Methods: This cross-sectional study was conducted among postdoctoral dental residents at the School of Dental Medicine, University of Nevada, Las Vegas. This cross-sectional study used images of cases of MIH and a survey to collect the data. The survey included demographics, educational background, and basic knowledge of MIH. Results: The response rate to the invitation to participate was 91%. The confidence in identifying MIH was 100%, 50%, and 33.3% for pediatric, orthodontic, and general practice residency (GPR). A total of 70% were aware of this anomaly from their predoctoral dental education and indicated the need for further related education. There was 33% confusion with fluorosis and 16.6% with amelogenesis imperfecta. A total of 66.6% of the participants indicated that they require further education relating to MIH. Conclusion: Within the limitations of the present investigation, MIH awareness among the investigated groups varied but was highest amongst the pediatric residents.

## 1. Introduction

While caries incidence is seemingly decreasing in developed countries, other oral disorders are becoming more prevalent. Molar incisor hypomineralization (MIH) has emerged recently as one of these disorders. The literature describes it as the presentation of an enamel defect, where incisors and one (or more) molars are affected [1]. Recently, Bussaneli et al., 2021, proposed that MIH is a qualitative, complex developmental defect of enamel (DDE) of multifactorial origin with a strong genetic component that affects at least one permanent molar and can often affect permanent incisors [2]. In this enamel malady, the dysfunction of the amelogenesis process underpins the occurrence of this phenomenon. In 2016, MIH global incidence was estimated to be 17.5 million people [3]. A recent comprehensive analysis of the prevalence reported it as quite common, with the highest prevalence in South America, with an estimate of approximately 14 percent [4]. Its prevalence has been linked to several etiological factors that correlate strongly, particularly prenatal illnesses during pregnancy or smoking. Perinatal issues such as prematurity, low birth weight or prolonged birth, cesarean delivery, and birth complications have been linked to MIH. Postnatal early childhood illness or medication, developmental determinants, ecological elements, and genetic components also appear to be factors in MIH development.

MIH has been shown to affect individuals’ quality of life and increases the potential of “negative self-perceptions of oral symptoms among children” [5]. Additionally, systemic exposures, such as maternal psychological stress respiratory diseases, are linked with higher odds of MIH, which is also significantly associated with fevers during the first years of a child [6]. A poorer oral health-related quality of life (OHRQoL) has been observed in children with moderate and severe MIH than those with mild or no MIH [7]. The negative effect of MIH not only affected the children but also the quality of life of their families [8].

MIH presents significant diagnostic and treatment challenges. Documented evidence of MIH in ancient populations indicates its etiological factors have been present for many centuries [9]. There is also the potential for hypersensitivity, difficulties with anaesthesia and anxiety, carious lesions, and the potential failure of restorations [10].

Initial and cavitated caries lesions have been associated with MIH [11]. It has been reported that MIH significantly affects caries development, being more common in hypomineralized molars. It is also evident that its associated caries prevalence is higher in children with MIH [12,13]. In addition to having a significant effect, it is also a challenge to manage for many practitioners. Therefore, exploring clinicians’ awareness, the ability of its identification and knowledge of its management are essential in providing efficient and high-quality oral health care to those affected. MIH diagnosis might be challenging due to its confusion with fluorosis, and it thus may be reported with fluorosis. This has been the case particularly in drinking water areas with moderate to high fluoride levels [14,15,16,17]. The severity levels of these two dental abnormalities seem to have a direct relationship; however, treatment approaches vary significantly. The challenge of its diagnosis has prompted numerous studies to evaluate clinicians’ perceptions and knowledge to solve these challenges; particularly that lower awareness levels and ability to diagnose MIH have been previously reported [15].

Several questionnaire-based studies regarding MIH have been conducted; the results of these studies show confusion regarding its prevalence, etiology, and treatment options [18,19,20]. An additional challenge is that approximately one-quarter of children with MIH will require interventions due to symptoms or post-eruptive tissue breakdown [3]. Such management varies according to how severe the hypomineralization is and the number of teeth affected. The null hypothesis is that the discipline residency has no effect on awareness and confidence in identifying and managing MIH.

This study evaluates the awareness and confidence in identifying and managing MIH in a cohort of residents.

## 2. Materials and Methods

Postdoctoral dental residents from orthodontic, pediatric, and GPR were invited to participate in this study. Participation was voluntary, informed written consent was duly obtained, and anonymity and confidentiality were assured.

This was a decriptive cross-sectional study. The survey used in this study was adopted and adapted (with permission) from Tagelsir et al., 2018 [17]. This survey included a MIH-related series of photographs that were provided to the participants and also included the demographics, their educational background, basic knowledge of MIH, enamel defects, prevalence, severity, differential diagnoses, and possible management (glass ionomer, compomer, amalgam, resin modified glass ionomer, composite resin, stainless steel crowns, cast restoration). The study received exempt status from the Institutional Review Board (IRB) of the University of Nevada at Las Vegas (UNLV)(#1487461).

Sample:

The inclusion criteria were orthodontic, pediatric, and GPR residents at the University of Nevada at Las Vegas, School of Dental Medicine.

After compiling the data from the survey, it was tabulated, analyzed, and statistically evaluated. Chi-square tests were used to interpret the results of any association between respective variables. The Lambda tests were applied for associations for the remainder of the sample.

## 3. Results

**Figure 1:**
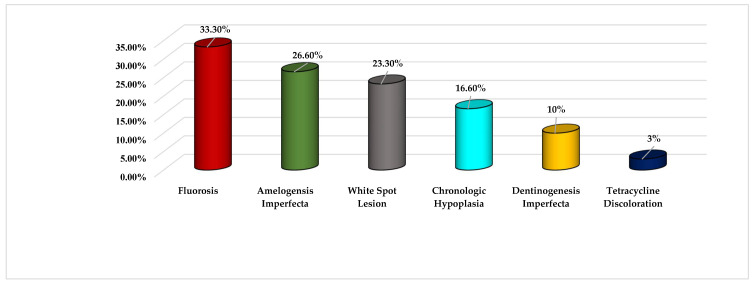
Participants’ choices for alternative diagnoses of MIH cases.

Out of the 33 residents, 30 completed the survey (9 pediatric dentistry, 17 orthodontics, and 4 general practice residents), with an overall response rate of 91%. MIH was confused with other anomalies; in 33% as fluorosis; 26.6% as amelogenesis imperfecta; 23.3% as white spot lesions; 16.6% as chronological hypoplasia; 10% as dentinogenesis imperfecta; and in 3% it was confused with tetracycline staining (Figure 1).

**Figure 2:**
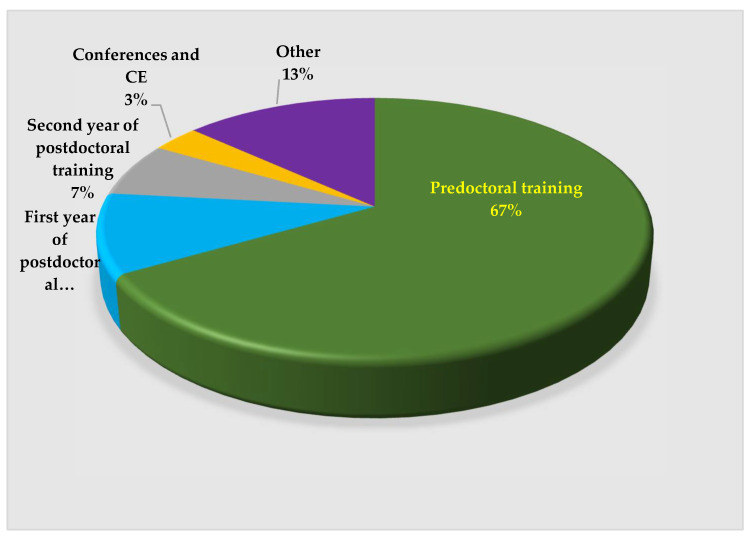
Residents’ recall of first learning about MIH.

The participants’ awareness of MIH was high, with 70% reporting having had previous exposure to this phenomenon. Figure 2 shows that 67% of the postdoctoral residents recall that they first learned about MIH during their predoctoral training.

When participants were asked about the need for further education relating to MIH, 66.6% indicated that this was required. There was a highly significant association between the participants’ related disciplines (orthodontics, pediatrics, general practice) and the level of confidence in identifying MIH. Pediatric residents were the most confident in their ability to identify this enamel abnormality (Table 1). They reported confidence in identifying MIH at 100%, with confidences of 50% and 33.3% for orthodontic and GPR, respectively.

The participants’ identification of MIH on the images and their recommended management with the restorative material of choice showed statistical significance in the first two case image scenarios and no significant association for the third scenario (Table 2).

## 4. Discussion

The increased awarness and research interest in MIH makes it one of the important subjects in modern dentistry. The present investigation found that the awareness of MIH varied among residents but was highest among pediatric residents. A similar trend has been reported among Hong-Kong dental care providers [21]. In another similar study, significant disparities in knowledge and management of MIH were found between dental practitioners in France [22]. When comparing the present results concerning the postdoctoral residents’ confidence about diagnosing MIH and the challenges in identifying MIH from other conditions, it seems that orthodontics and general practice postdoctoral residents were less confident in identifying MIH and had some difficulty in distinguishing MIH from other conditions. Craveia et al. found that general dentists and orthodontists misdiagnosed MIH [22]. For the pediatric postdoctoral residents, similar to what was reported by Tagelsir et al. they were more confident in identifying MIH [17]. The element of novelty in this study, compared to the studies by Gamboa et al. (2018) [21] and that of Craveia et al. (2020) [22], is the evaluation of three groups of residents from three different disciplines.

It is essential to improve future dental professionals’ abilities to diagnose MIH early, since patients’ level of care and well-being would significantly improve. However, the challenge is in confidently determining MIH definitive diagnosis, particularly among younger children whose permanent teeth are still erupting, and where the distribution of any enamel defect may not be evident [20].

The effect of MIH could be enormous on children and adult patients, impacting on self-confidence and well-being [13]. Therefore, increasing the emphasis on MIH awareness and diagnosis in both predoctoral education and postdoctoral residency programs could be one way of improving early diagnosis amongst dental professionals [23,24,25]. The limited education and awareness of MIH has previously been reported; for example, 64% of predoctoral dental students in a Saudi Arabian study reported not having heard of this condition and favored including an MIH case-based teaching in their curriculum [24]. On the other hand, a recent study in Austria showed that 98% of the undergraduate dental students were familiar with MIH, and 86% were aware of its clinical presentation [26]. Therefore, evaluation of the level of awareness should be a feature in predoctoral dental students’ and postdoctoral residents’ education and assessments.

In addition to the wide variation in the clinical presentation, there is also a variation in the individual’s needs for management. The management of MIH is further challenged by the limited evidence-based information on what works best. Furthermore, the accurate identification of MIH is a significant factor in making the appropriate management decisions and avoiding pain or other consequences of this condition. There are extensive management modalities for MIH, ranging from a conservative procedure such as prevention restoration to a more aggressive approach such as extraction [27]. Treatment might also be more challenging in the case of children with intellectual disabilities. A recent study indicated that the presence of MIH is considered a common problem among children with intellectual disabilities [28]. Treatment options may include fluoride varnish, sealants, conventional and resin-modified glass ionomers, composite resin, preformed metal crowns, and in severe cases, extractions of permanent molars [27,29].

In the present study, the dental material of choice by participants was resin-modified glass ionomer, consistent with other studies [30,31]; however, it was not in agreement with a systematic review that indicated the material of choice for MIH is reported to be composite restoration and preformed metal crown [10].

As more recognition of the need for the ability to diagnose and manage MIH increases, it is anticipated that the need for education related to it will increase in future generations. Indeed, there was a request for training courses or continuous education voiced by this cohort, which was similarly requested in previous reports [17,21,30,32]. Furthermore, the variation in treatment choice among these postdoctoral residents also signifies the need for treatment guidelines for the MIH, which should play a role in ensuring appropriate treatment decisions.

One of the limitations is the reasonably small sample size in this study. However, while one German study had a significantly larger sample than in this study, their sample population was made up of undergraduate dental students [14] while ours, although smaller, was made up of a different population, which included all three postgraduate dental residencies at the school of Dental Medicine, University of Nevada, Las Vegas, with a response rate of 91%. While the sample was small, nonetheless differences between the groups was detected. Accordingly, the results may be less conclusive, and a larger confirmatory study would be required, which is one of the recommendations from this investigation. The study was also not meant to quantify outcomes in a large population of postgraduate residents but to document the level of awareness of MIH among this specific group [33]. The results of this study should also be viewed with the limitation that the identification of MIH was based on photographic images and not on clinical examination. Further larger sample studies are required to explore and improve awareness in order to develop consistent clinical management strategies.

## 5. Conclusions

Within the limitations of the present investigation, it was concluded that awareness of MIH varied among residents but was highest in the pediatric residents group. Further larger sample studies should consider the evaluation of the awareness and management approaches of MIH among various groups.

## Figures and Tables

**Table 1:** Association between the line of specialty and level of confidence in identifying MIH.

Group	Very Confident	Confident	Not Confident	*p*-Value
Pediatric (%)	44.4%	55.6%	0.0%	
Ortho (%)	0.0%	41.2%	58.8%	0.005
GPR (%)	0.0%	33.3%	66.7%	
Total (%)	13.8%	44.8%	41.4%	

**Table 2:** Residents’ preferable restorative materials to manage cases with MIH.

Cases	Restoration of Choice	Percent	*p*-Value
**Case 1:** *Healthy 9-year-old with MIH on a symptomless first permanent molar*	Glass ionomer	10%	
	Resin-modified GI	48%	0.012
	Composite resin	35%	
	Stainless steel crowns	7%	
**Case 2:** *Healthy 10-year-old MIH on first permanent molar with hypersensitivity*	Glass ionomer	4%	
	Resin-modified GI	7%	0.025
	Composite resin	20%	
	Stainless steel crowns	65%	
**Case 3:** *Healthy 4-year-old with MIH on a symptomless second primary molar*	Glass ionomer	12%	
	Resin-modified GI	50%	0.183
	Composite resin	26%	
	Stainless steel crowns	12%	

## Data Availability

All data have been included in this study.
